# Correction: Camel Whey protein ameliorates type Ⅰ diabetic cardiomyopathy by mitigating oxidative stress, inflammation and apoptosis

**DOI:** 10.1007/s44463-026-00092-7

**Published:** 2026-06-25

**Authors:** Zeyan Peng, Jingyang Wang, Zhili He, Ziwei Chen, Han Wei, Xiaowen Zhang, Zhiyong Shi, Zhiyong Wang, Liang Yang, Jie Yan, Jing Li, Jianlin Cui, LiFeng Feng, Ying Liu, HaiTao Yue, Kai-Chiang YANG, Zhi Qi, Jie Yang, Yang Gao

**Affiliations:** 1 Department of Molecular Pharmacology, School of Medicine, Nankai University, Tianjin, 300071 China; 2 College of Life Science and Technology, Xinjiang University, Urumqi, 830046 Xinjiang China; 3 Affiliated Hospital of Nankai University, Tianjin, 300222 China; 4 Tianjin Union Medical Center, Tianjin, 300122 China; 5 Xinjiang Camel Industry Engineering Technology Research Center, Urumqi, 830046 Xinjiang China; 6 School of Dental Technology, College of Oral Medicine, Taipei Medical University, Taiwan, 110301 China; 7 Nankai University Eye Institute, Tianjin, 300110 China; 8 Xinjiang Production and Construction Corps Hospital, Xinjiang, 830092 China

**Correction to: Food Science of Animal Resources (2026) 46:29**

10.1007/s44463-025-00021-0

In this article the abstract section has been omitted. It should read:

**Abstract:** Diabetic cardiomyopathy (DCM) is the leading cause of death in diabetic complications. Inflammation and oxidative stress greatly influence the progression of DCM and there are limited therapeutic opportunities. It has been shown that camel whey protein (CWP), a functional dairy ingredient has antioxidant and anti-inflammatory properties. Our objective is to illustrate the cardioprotective effect of CWP, a natural product in DCM and underlying mechanism of action. We found in type Ⅰ diabetic mice induced by STZ, oral intake of CWP significantly prevents cardiac hypertrophy. The biomarkers of inflammation, oxidative stress and cell apoptosis in heart tissue were also suppressed by CWP. Further, we determined the activation of antioxidant nuclear factor erythroid 2-related factor 2 (Nrf2)/heme oxygenase-1 (HO-1) signaling pathway in regulating this molecular niche. By using neonatal rat ventricular myocytes (NRVMs) treated with high glucose, we found that CWP protects cells from oxidative stress and cell apoptosis. Our results suggested that CWP could serve as a clinical nutritional supplement in the prevention of DCM due to its function of suppressing inflammation, oxidative stress and cell apoptosis. 

The original article has been corrected.

